# High *p*CO_2_-induced exopolysaccharide-rich ballasted aggregates of planktonic cyanobacteria could explain Paleoproterozoic carbon burial

**DOI:** 10.1038/s41467-018-04588-9

**Published:** 2018-05-29

**Authors:** Nina A. Kamennaya, Marcin Zemla, Laura Mahoney, Liang Chen, Elizabeth Holman, Hoi-Ying Holman, Manfred Auer, Caroline M. Ajo-Franklin, Christer Jansson

**Affiliations:** 1 Earth Sciences Division, Lawrence Berkeley National Laboratory (LBNL), Berkeley, CA 94720 USA; 2 Molecular Biophysics and Integrated Bioimaging Sciences Division, LBNL, Berkeley, CA 94720 USA; 3 Department of Chemistry, University of California, Berkeley, CA 94720 USA; 4 The Molecular Foundry, LBNL, Berkeley, CA 94720 USA; 5 Present Address: School of Plant Sciences and Food Security, The George S. Wise Faculty of Life Sciences, Tel Aviv University, Tel Aviv, 6997801 Israel; 6 Present Address: The Environmental Molecular Sciences Laboratory, Pacific Northwest National Laboratory, P.O Box 999, K8-93, Richland, WA 99352 USA

## Abstract

The contribution of planktonic cyanobacteria to burial of organic carbon in deep-sea sediments before the emergence of eukaryotic predators ~1.5 Ga has been considered negligible owing to the slow sinking speed of their small cells. However, global, highly positive excursion in carbon isotope values of inorganic carbonates ~2.22–2.06 Ga implies massive organic matter burial that had to be linked to oceanic cyanobacteria. Here to elucidate that link, we experiment with unicellular planktonic cyanobacteria acclimated to high partial CO_2_ pressure (*p*CO_2_) representative of the early Paleoproterozoic. We find that high *p*CO_2_ boosts generation of acidic extracellular polysaccharides (EPS) that adsorb Ca and Mg cations, support mineralization, and aggregate cells to form ballasted particles. The down flux of such self-assembled cyanobacterial aggregates would decouple the oxygenic photosynthesis from oxidative respiration at the ocean scale, drive export of organic matter from surface to deep ocean and sustain oxygenation of the planetary surface.

## Abstract (web summary)

A Paleoproterozoic carbon isotope anomaly is likely linked to burial of oceanic cyanobacteria, but it is not clear how burial occurred. Here, the authors find that, under Paleoproterozoic *p*CO_2_ conditions, planktonic cyanobacteria increase exopolysaccharide production and mineralization, leading to aggregation and faster sinking.

## Introduction

The process of oxygenic photosynthesis evolved in cyanobacteria in a mildly reduced environment of the late Archean Eon^1–6^. Owing to the ubiquity of its electron source H_2_O, oxygenic photosynthesis was superior to alternative metabolic pathways^7, 8^ and, by further inhibiting some of them through generation of O_2_, oxygenic photosynthesis monopolized the available sun-lit environments^9^ and drove Earth’s surface oxygenation (the great oxygenation event, GOE) ~2.3 Ga^2, 10^. The oxygenation was followed by global, highly positive, excursion in carbon isotope values of inorganic carbonates (δ^13^C) (the ~2.22–2.06 Ga Lomagundi–Jatuli carbon isotope anomaly)^11^, which reflects enrichment of seawater carbonate pool in ^13^C plausibly caused by increased organic matter burial^12^. This extraordinary anomaly (δ^13^C up to 28‰) suggests extreme rates of production and burial of the isotopically lighter organic carbon in oceanic interior and sediments at a planetary scale^13^.

Sequestration of organic carbon in Paleoproterozoic (2.5–1.6 Ga) shallow water environments is evident from a robust fossil record of mat-forming (cyano)bacteria preserved in the shallow water biosediments, aka stromatolites^14–16^. However, only a relatively small area of ocean floor received enough light for stromatolite-associated photosynthesis. Therefore, the biomass produced by benthic mat-forming cyanobacteria, which is all the more prone to re-mineralization rather than burial, appears insufficient to account for the global C isotope anomaly. Hence, with ≥75% of the Paleoproterozoic planet covered by oceans^17^, and assuming negligible burial of terrestrial organic matter, oceanic planktonic unicellular cyanobacteria ought to contribute to the global sequestration of organic carbon.

Both phylogenomic and microfossil evidences^18, 19^ suggest that unicellular cyanobacteria were present during the Paleoproterozoic, though their planktonic growth could be restricted by ultraviolet (UV) radiation until it was sufficiently screened by atmospheric O_2_ and O_3_^20, 21^. Furthermore, because of slow sinking rate of their small cells, the organic matter of planktonic cyanobacteria should not sediment before the emergence of eukaryotic predators ~1.5 Ga^22^ (aka biological carbon pump^23^). It is assumed that without predator-mediated repackaging of the small unballasted cell particles to denser aggregates, single cells were remineralized before reaching deep waters^24, 25^. Therefore, the mechanism of carbon sequestration, which led to the Lomagundi–Jatuli carbon isotope anomaly remains unexplained.

Owing to tectonic reworking of oceanic crusts, little fossil record of the pelagic organic carbon sequestration remains to explore^26^. Therefore, to understand how ancient planktonic cyanobacteria could bury their own biomass, we used an alternative experimental approach by capitalizing on physiological plasticity of living cyanobacteria, which plausibly derived from their evolutionary genetic memory. Here we test the hypothesis that planktonic unicellular cyanobacteria themselves could drive the export of organic carbon in the Paleoproterozoic open ocean.

Oxygenic photosynthetic cyanobacteria fix carbon to generate cellular biomass as well as EPS that create microenvironment around the cyanobacterial cells. EPS in cyanobacteria is characterized by high content of negatively charged (acidic) groups, e.g., carboxylic group (R–COO^–^) of uronic acids and pyruvate, phosphoryl (R–PO_3_^−^), sulfate (R–SO_4_^−^), and sulfonate (R–SO_3_^−^) moieties^27^. Therefore, cyanobacterial EPS can effectively bind and concentrate mono- and divalent metal cations (refs. ^28, 29^ and references therein), while multivalent metals can form cation bridges between the acidic EPS groups, conferring EPS adhesive and flocculating capacity^30–32^. Depending on a spatial distribution of the negative charges, the organic matrix of the EPS was also shown to either facilitate mineral nucleation, or effectively inhibit it by complexing Ca and Mg cations^28, 33^. More generally, the EPS matrix can protect a cyanobacterial cell from its environment by creating a mechanical and a diffusion barrier and by accumulating compounds that protect from excessive illumination and harmful UV radiation^29^.

Growth of cyanobacteria depends on availability of C, nitrogen (N), and phosphorus (P) in amounts sufficient for synthesis of the organic matter with approximate C:N:P atomic ratios of 106:16:1, respectively^34^. Nitrogen fixation, that probably predated oxygenic photosynthesis^35^ but was acquired by cyanobacteria early in their evolution^36^, relieved the iron-rich Paleoproterozoic Ocean from N deprivation^37^. Recurrent glaciation–deglaciation cycles as well as groundwater acidification facilitated continental weathering and fueled the Paleoproterozoic oceans with phosphorus^38, 39^. Finally, availability of inorganic carbon, the principal C source for cyanobacteria that is proportional to *p*CO_2_ in the atmosphere, was much higher than at present. The present atmospheric level (PAL, 4 × 10^−4^ bar) of CO_2_ actually limits the photosynthetic activity in modern cyanobacteria and, to compensate for that, they have evolved a suit of auxiliary mechanisms that jointly elevate the intracellular CO_2_ concentration to the level sufficient for oxygenic photosynthesis^40^. This carbon-concentrating mechanism (CCM) includes a series of bicarbonate and CO_2_ transporters, which become dispensable at high *p*CO_2_ and are at least partially inhibited^41^. Ancient cyanobacteria plausibly lacked CCM owing to the high Paleoproterozoic levels of *p*CO_2_ that were sufficient to support the oxygenic photosynthesis^40, 42^.

While the exact ancient *p*CO_2_ values are still debated, the ultrahigh Neoarchaean *p*CO_2_, initially estimated to 100–600 times above the PAL, were required to keep the Earth surface above freezing with solar luminosity 20–30% lower than at present^43, 44^. On the other hand, ambient CO_2_ concentrations inferred from weathering profiles^45–47^ pointed to lower *p*CO_2_, suggesting presence of additional greenhouse gases, e.g., methane^48^, in the Archean atmosphere. Recent estimates of *p*CO_2_ deduced from chemical composition of paleosols are high (0.034–0.244 bar) in Neoarchaean (2.8–2.5 Ga) with short-term fluctuations but a general trend of gradual decrease to 0.0092–0.084 bar toward the end of Paleoproterozoic (1.85 Ga)^49^. Guided by these data, we choose 0.05 bar and 0.15 bar *p*CO_2_ as the low and the high estimated level of CO_2_ in the Paleoproterozoic atmosphere of 2.3–2 Ga. We use microscopy and qualitative as well as quantitative analytical methods to examine morphological and physiological properties of *Synechococcus* sp. strain PCC8806 (*S*. 8806)^50, 51^ grown at the chosen CO_2_ levels. Under these conditions, the cyanobacteria generate copious amounts of EPS, which aggregate cells, adsorb metal cations, and support nucleation of amorphous minerals, thereby forming ballasted cell aggregates with high sinking potential. In the Paleoproterozoic, formation of such ballasted cell aggregates could have resulted in efficient organic carbon export from the surface to deep ocean by unicellular planktonic cyanobacteria.

## Results

### The model for Paleoproterozoic planktonic cyanobacteria

To grow cyanobacteria under the ultrahigh levels of CO_2_ estimated for Paleoproterozoic, we used a single-cell marine cyanobacterium as a model because of its planktonic growth and resilience to high *p*CO_2_. The *S*. 8806 cultivated with 4 × 10^−4^ bar *p*CO_2_ (henceforth referred to as PAL control) was transferred to 0.05 bar *p*CO_2_ and then gradually acclimated to 0.15 bar *p*CO_2_ until growth at a constant rate was achieved (Table 1). Expression of two bicarbonate transporters common to CCM of marine cyanobacteria^52^ was strongly downregulated (4.5 times for SbtA and 19 times for BicA) in the latter compared to the PAL control, suggesting that under high *p*CO_2_ *S*. 8806 downshifts its CCM and relies primarily on diffusion of CO_2_ for oxygenic photosynthesis as did the ancient cyanobacteria^40, 42^. The *S*. 8806 cells, cultivated at 0.05 or 0.15 bar *p*CO_2_, are thus further referred to as the low Proterozoic and the high Proterozoic models, respectively.

**Table 1:** Characteristics of *S*. 8806 cells cultivated under different *p*CO

*S*. 8806 culture (*p*CO_2_ bar)	Cell diameter	SGR	Chl *a*	PC
PAL control (4 × 10^−4^)	2.26 ± 0.41	0.48 ± 0.12	14.8 ± 2.7	42.2 ± 15.4
Low Paleoproterozoic (0.05)	2.28 ± 0.17	0.56 ± 0.02	23.5 ± 3.5	113.0 ± 26.9
High Paleoproterozoic (0.15)	2.36 ± 0.57	0.45 ± 0.08	26.4 ± 3.9	140.4 ± 35.7

Cell diameter (μm), specific growth rate (SGR, d^−1^), chlorophyll *a* (Chl *a*), and phycocyanin (PC) cell content (μg mg^−1^ soluble protein) of *S*. 8806 cultures cultivated under different *p*CO_2_. The range given is ±s.d., with *n* = 12 for cell diameter, *n* = 4 for SGR, and *n* = 3 for Chl *a* and PC.

### Increased EPS generation by the model cyanobacteria

**Fig. 1:**
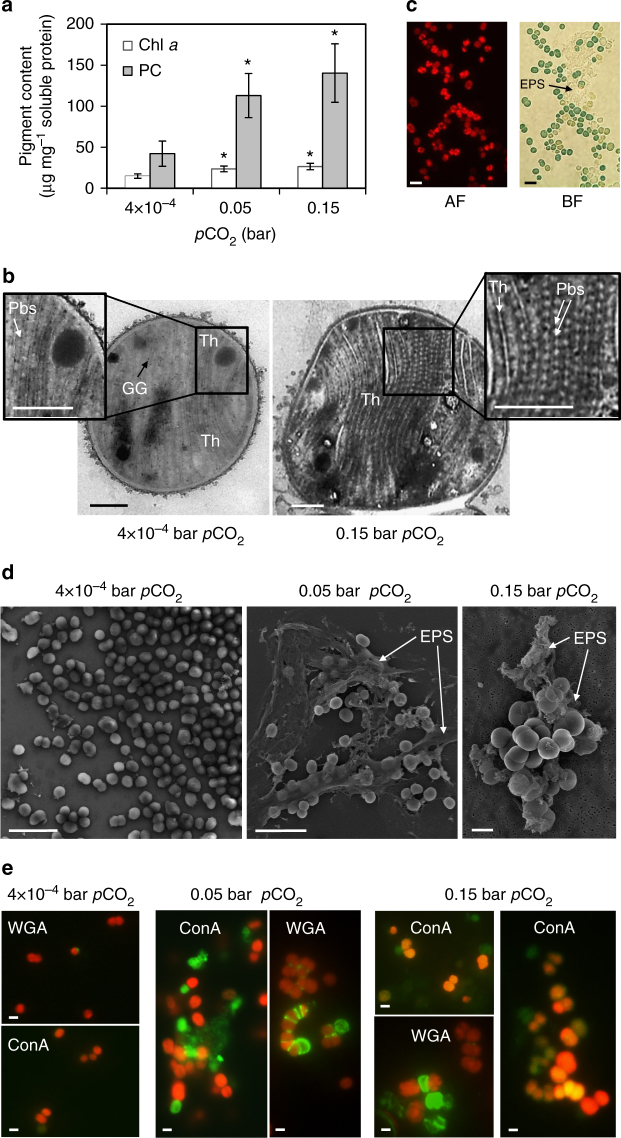
Physiological, morphological, and ultrastructural characteristics of *S*. 8806 cells. **a** Concentrations of photosynthetic pigments chlorophyll *a* (Chl *a*) and phycocyanin (PC) in *S*. 8806 increased with increase in *p*CO_2_. Data are mean ± s.d., *n* = 3 (biological replicates). * indicates statistically significant difference as compared to the PAL control (*t*-test, *p* < 0.05, Supplementary Table 1). **b** Thin-section transmission electron microscopy (TEM) micrographs reveal more compact packing of phycobilisomes (Pbs)-bearing thylakoid membranes (Th) in the high Proterozoic model cells compared to cells from the PAL control. No pronounced increase in intracellular accumulation of glycogen granules (GG) is noticeable in the Proterozoic model cells. Scale bar = 0.5 μm. **c** The high Proterozoic model cells visualized in an epifluorescence mode under green excitation have red chlorophyll autofluorescence (AF). A bright-field (BF) mode image reveals that the cells are embedded in a semi-transparent EPS matrix and form aggregates, characteristic of the Proterozoic model cultures. Scale bar = 5 μm. **d** The SEM micrographs show abundant EPS associated with cells of the Proterozoic model but not with the PAL control cells. Scale bar = 10 μm. **e** False-color EPS stains (WGA and ConA; 488 nm line, FITC filter, green) imposed over cyanobacterial cells (chlorophyll autofluorescence; 594 nm line, Texas Red filter, red) show very little stain signal for the PAL control cells but dramatic accumulation of mannosyl, glucosyl (ConA), and *N*-acetylglucosamine residues (WGA) in cell aggregates from the Proterozoic model cultures. Bar scale = 1 μm. See Supplementary Fig. 2 for single-line images

Spectrophotometric analyses showed that mean concentration of the reaction center pigment chlorophyll *a* in low and high Proterozoic model cells were 1.6 and 2.7 times higher, respectively, than in the PAL control cells (*t*-test, *p* < 0.05). Similarly, a 1.8 and 3.3 times increase was measured for photosynthetic antenna pigment phycocyanin (*t*-test, *p* < 0.05) (Fig. 1a, Table 1, and Supplementary Table 1). The Proterozoic model cells showed tight arrangement of the thylakoid membranes bearing densely packed light-harvesting phycobilisome complexes (Fig. 1b and Supplementary Fig. 1). The increased content of photosynthetic complexes in Proterozoic model cells suggested that, compared to the PAL control, the cyanobacterial cells exposed to high *p*CO_2_ should have higher rates of photosynthetic CO_2_ fixation and thence higher cell growth. Yet, both the size and the division rate of Proterozoic model cells were not significantly different from those of the PAL control (Table 1 and Supplementary Table 1). Furthermore, no noticeable accumulation of glycogen granules in cells of the Proterozoic models was evident (Fig. 1b). These observations raised a question about the metabolic fate of the excess carbon assimilated by the Proterozoic models. We noticed abundant formation of mucilage-rich cell aggregates, which settled to bottom of flasks containing the Proterozoic model cultures (Fig. 1c), while PAL control cells remained dispersed and were associated with no prominent mucilaginous investments in agreement with the general genus-specific morphological characteristics^51, 53^. This observation suggested that the Proterozoic model cells excreted the assimilated excess carbon in a form of polysaccharides. Indeed, immunostaining and microscopic analyses confirmed the presence of copious carbon-rich EPS in association with cells of the Proterozoic models (Fig. 1d, e and Supplementary Fig. 2).

### Cation sorption by acidic EPS of Proterozoic model cells

**Fig. 2:**
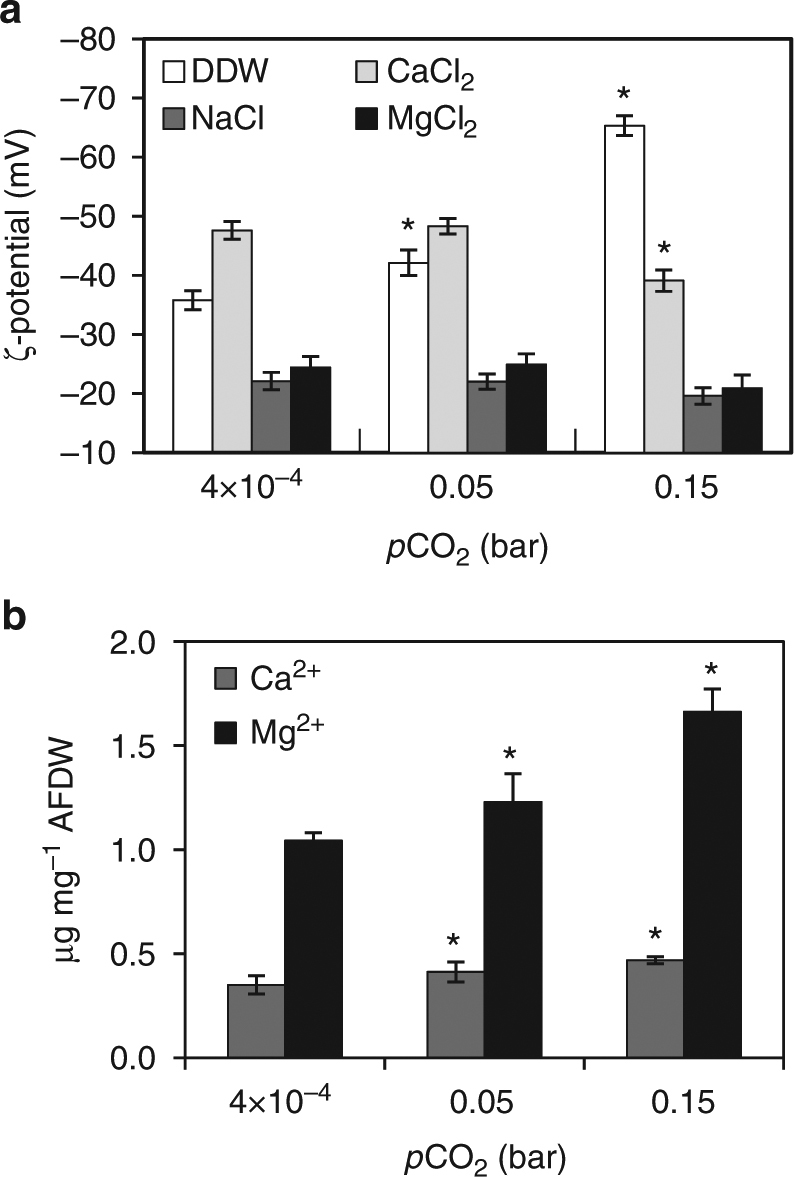
Cell surface electronegativity and ion retention capacity of *S*. 8806 cells. **a** The ζ-potential (mV) of cells suspended in DDW was significantly more negative for the Proterozoic model cultures compared to cultures of the PAL control. The differential ζ-potential in the Proterozoic model cells could be screened by either CaCl_2_ or MgCl_2_ solutions but not by NaCl. Data are mean ± s.d., *n* = 3 (biological replicates). * indicates statistically significant difference as compared to the PAL control (*t*-test, *p* < 0.05, Supplementary Table 2). **b** High cell surface electronegativity conferred cells from the Proterozoic model cultures with capacity to retain more ions compared to cells of the PAL control. Data are mean ± s.d., *n* = 3 (biological replicates). AFDW ash-free dry weight; * indicates statistically significant difference as compared to the PAL control (*t*-test, *p* < 0.05, Supplementary Tables 1 and 3)

Since the negative charges of cyanobacterial EPS can facilitate cell flocculation and metal binding, we assessed the effect of EPS accumulation on the surface charge of Proterozoic model cells. We determined the degree of cell peripheral electronegativity by measuring zeta- (ζ-) potential that derives from electrophoretic mobility of cells in a solute^54^. The ζ-potential of −65 ±2 mV measured for the high Proterozoic model cells dispersed in distilled water was almost double that of the value measured for the PAL control (Fig. 2a and Supplementary Table 2). That shift in ζ-potential reflected the increase in peripheral negativity of the high Proterozoic model cells (Fig. 1d, e) conferred by copious EPS. The intermediate value of −42 ±1 mV measured for low Proterozoic model indicated that accumulation of acidic EPS was proportional to CO_2_ availability (Fig. 2a). We then measured the capacity of Proterozoic model cells to bind Na, Ca, and Mg cations abundant in the present day as well as in the Proterozoic seawater. Addition of Na^+^ (as NaCl), the most abundant cation of seawater, only partially screened the highly negative ζ-potential of the high Proterozoic model cells (Fig. 2a and Supplementary Table 2). However, addition of Ca^2+^ and Mg^2+^ (as CaCl_2_ and MgCl_2_) converged ζ-potentials of all cell types to −22 ±2 mV, screening as much as two-thirds of the negative ζ-potentials in the high Proterozoic model cells (Fig. 2a). Therefore, the increased peripheral electronegativity, caused by *p*CO_2_-dependent EPS accumulation, attracted metal dications in preference to monocations.

To directly quantify sorption of Ca^2+^ and Mg^2+^ by EPS associated with the Proterozoic model cells, we measured removal of the cations from a solution by metabolically inactivated cells. We observed >30% increase in Ca^2+^ and 60% increase in Mg^2+^ removal for the high Proterozoic model compared to the PAL control cells (Fig. 2b and Supplementary Table 3). Intermediate values measured for cells of the low Proterozoic model (Fig. 2b) agreed with the notion that the capacity of cells to adsorb dications was proportional to CO_2_ availability. In total, weight of Ca and Mg cations in cells of the high Proterozoic culture increased >1.5 times compared to the PAL control culture, raising the cells’ density by at least 0.07% (Supplementary Table 3). Visually, the Proterozoic model cells suspended in NaCl solution flocculated upon addition of Ca and Mg dications and then settled contrary to the PAL control cells that remained dispersed in the solution.

### Accumulation of cations and amorphous minerals in EPS matrix

**Fig. 3:**
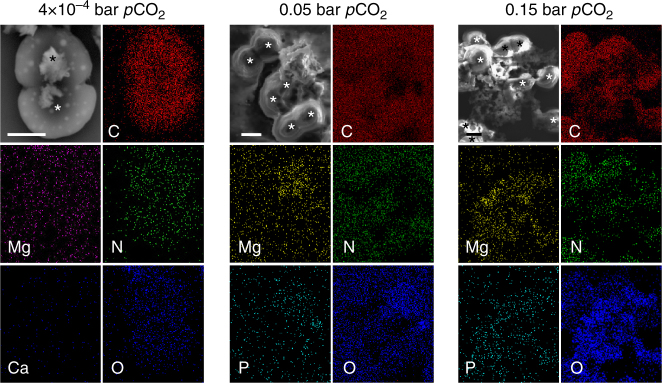
Accumulation of Mg in EPS of the Proterozoic model culture. The SEM-coupled EDS elemental analysis showed no distinct accumulation of either Ca or Mg elements in the PAL control cells but revealed accumulation of Mg in EPS matrix of cell aggregates from the high Proterozoic model cultures. The EPS, i.e., carbohydrates, are represented by an overlap between C and O, an overlap between C, O, and N signals represents proteinaceous cells (*) and an overlap between Mg and P signals in EPS of the Paleoproterozoic cell aggregates suggests the presence of Mg–P mineral salts. Scale bar = 1 μm

To check whether increased dication sorption by the Proterozoic model cells resulted in increased formation of minerals, we searched for crystalline solids. Scanning electron microscopy (SEM) micrographs showed no characteristic mineral crystals that were associated with cells of any of the cultures (Fig. 1d). On the other hand, SEM-coupled energy dispersive X-ray spectroscopy (EDS) elemental analyses revealed increased content of Mg in cell aggregates of the Proterozoic model compared to the PAL control cells (Fig. 3), in agreement with our previous results (Fig. 2). The Mg clusters could be mapped to EPS matrix rather than to cells in the Proterozoic cell aggregates. Repeatedly, Mg signal coincided with signals of oxygen, phosphorus (P), and chlorine (Cl) (Fig. 3 and Supplementary Fig. 3), implying presence of Mg hydroxides, phosphates, and chlorides. However, powder X-ray diffraction analysis revealed no informative peaks in dried biomass of the Proterozoic model cells, suggesting that these minerals were amorphous in bulk X-ray diffraction. Therefore, we concluded that in aggregates of the Proterozoic model cells EPS matrix concentrated Ca and Mg cations that could form amorphous mineral phases with a variety of seawater anions.

## Discussion

Physiological plasticity of cyanobacteria (e.g., downregulation of CCM), which potentially represents the evolutionary memory encrypted in their genetic material, enabled *S*. 8806 to adapt to ultrahigh *p*CO_2_ experienced by ancient cyanobacteria. The increased photosynthetic potential in the Proterozoic model cells (Fig. 1) is in agreement with observations of increased density of active reaction centers and enhanced biomass production in green alga *Chlorella minutissima* cultivated with similar *p*CO_2_^55^. In the Paleoproterozoic model cultures CO_2_, which was fixed in excess and not utilized by cellular metabolism, was shunted to synthesis of saccharides, which were then exuded. We assume that massive generation of EPS, also referred to as the “overflow metabolism”^56, 57^, was triggered by excess availability of C relative to other macronutrients (N and P) and was essential for physiological adaptation of modern cyanobacteria to high *p*CO_2_. On the other hand, because the “overflow metabolism” is generally common to cyanobacteria and microalgae^57–60^, it could be inherently characteristic of photosynthetic systems and have an ancestral root. Owing to the ultrahigh atmospheric CO_2_ levels of the Paleoroterozoic era, C availability to the ancient planktonic cyanobacteria was disproportionally high relative to N and P availability^61–63^. We suggest that this high relative availability of CO_2_ similarly stimulated excess generation of EPS in Paleoproterozoic planktonic cyanobacteria.

The EPS-rich Proterozoic model cells remained dispersed in NaCl solution but flocculated in artificial seawater (Fig. 1c, d, e) or when Ca^2+^ and Mg^2+^ were added to NaCl solution. A well-documented adhesive quality of fibrous EPS matrix is conferred by its polar constituents^27, 32^. When Ca and Mg dications form cross-bridges between the acidic EPS groups, they flocculate the EPS and consequently EPS-associated cells^31^. Similarly, negatively charged EPS excreted by Paleoproterozoic planktonic cyanobacteria should flocculate them. Multivalent cations of Fe are even more effective in crosslinking negatively charged microalgal cells^30^ and could further facilitate flocculation of EPS-rich planktonic cyanobacteria in a yet-patchily reduced Paleoproterozoic ocean.

The density (g cm^−3^) of EPS-bound aggregates of the Proterozoic model cell is higher than the density of dispersed single cells in the PAL control culture. Accumulation of Ca^2+^ and Mg^2+^ in EPS matrix of the Proterozoic model cell aggregates further increases their density, ballasting the aggregates by at least 0.07% (Fig. 2b and Supplementary Table 3). This is a conservative minimal estimate because it does not account for counter anions (e.g., HPO_4_^3−^, Cl^−^, or OH^−^), which form mineral salts with Ca/Mg cations. Assuming that the density of the neutrally buoyant PAL cells equals the density of seawater—1.0219 g cm^−3^ (with 33‰ salinity at 25 °C), the estimated density of the Paleoproterozoic cells is at least 1.0226 g cm^−3^. That was sufficient for the aggregates of Paleoproterozoic model cells to settle down to a flask bottom. Because sorption of Ca^2+^, Mg^2+^, and other metal cations by cyanobacterial EPS is universal for present-day cyanobacteria^29^, then similar ballasting of cyanobacterial biomass should happen in the Paleoproterozoic Ocean. Faster sinking of such ballasted cell aggregates below the oxygenated water layer would reduce oxidative respiration of organic matter and consequently intensify burial of the organic matter in marine sediments^24, 64^.

Extreme rates of organic matter burial in the Paleoproterozoic Ocean, recorded in the highly positive Lomagundi δ^13^C excursion^11^, were essential for sustaining the Earth’s surface oxygenation. Furthermore, recently suggested Proterozoic oxygen regulation^65^ explains how upsurge in the rate of organic carbon burial could trigger Earth’s surface oxygenation without leaving a trace in the long-term δ^13^C record. Hence, expansion of planktonic cyanobacteria into the Open Ocean ~2.3 Ga^19^ could drive the GOE through the proposed mechanism for organic matter burial applied at a planetary scale.

Excess generation of EPS could be essential for expansion of the Paleoproterozoic cyanobacteria into the Open Ocean. While benthic cyanobacteria could control their microenvironments in semi-enclosed microbial mats of ancient shallow oceans^66^, unicellular planktonic cyanobacteria could create a protective EPS buffer, which would shield them from a hostile environment. For example, harmful interactions between Fe^2+^ and oxygen^67^ could be attenuated by a diffusion barrier of highly hydrated EPS^32^, moving the reactive boundary away from the cell surface and thereby protecting the cell from oxidative damage. Similarly, immobilization by EPS of toxic heavy metals would prevent them from reaching the cell^68, 69^. Finally, accumulation of UV-screen pigments and retention of metal cations and Fe-oxides in the EPS matrix could shield the ancient planktonic cell from harmful UV radiation.

In summary, unicellular planktonic cyanobacteria acclimated to Paleoproterozoic ultrahigh CO_2_ levels generated copious EPS that resulted in flocculation of the model cells and in formation of ballasted aggregates. We propose that the ballasted self-assembled cyanobacterial aggregates could provide the missing conduit for extensive export of organic carbon from the surface to deep Paleoproterozoic Ocean.

## Methods

### Organisms and culture conditions

Single-cell sheathless marine cyanobacterium *S*. 8806 (Cyanobacterium genus)^51, 53^, obtained from the American Type Culture Collection, was cultivated as a semi-batch culture in modified artificial seawater ASNIII^70^ containing 8.8 mM NO_3_^−^, 5–10 mM Ca^2+^, 24 mM Mg^2+^, and 0.09 mM PO_4_^2−^. The culture was maintained at 25 °C with orbital agitation of 60 rpm in continuous light of 50 ± 10 μmol photons m^−2^ s^−1^ (New Brunswick Innova® 44R), aerated with humidified air containing 4 × 10^−4^ bar *p*CO_2_ (PAL) at a rate of 1 L gas min^−1^ (L culture)^−1^, and sub-cultured weekly. To develop the Proterozoic model cyanobacteria, *S*. 8806 cells were first transferred to air-balance gas mixture of 0.05 bar *p*CO_2_ (125 PAL) for ten generations. The resulting culture was subjected to gradual increase in *p*CO_2_ until it tolerated 0.15 bar *p*CO_2_ (325 PAL) introduced at a rate of 1 L gas min^−1^ (L culture)^−1^. We considered physiological acclimation to 0.15 bar *p*CO_2_ to be completed after stabilization of the culture growth rate (Table 1).

### Pigment content determination

For quantitative determination of cell pigments, chlorophyll *a* from cell pellets of 1 ml from the late mid-log phase culture (OD_750_ ~1.0) was extracted overnight in dark at +4 °C with 100% methanol. To determine phycocyanin content, 2 ml of the culture were harvested by centrifugation, resuspended in 1 ml of phosphate buffer saline (10 mM, pH 7.2), and disrupted with glass beads (0.1 mm) using a FastPrep®-24 beadbeater (MP Biomedicals, www.mpbio.com). Subsequently, the samples were centrifuged at maximal speed and the supernatant containing the extracted water-soluble pigments was collected. The concentrations of chlorophyll *a* and phycocyanin were estimated spectrophotometrically (DU 640 Spectrophotometer, Beckman Coulter Inc., www.beckmancoulter.com) and calculated as [Chl *a*] = 16.72 × OD_665_ − 9.16 × OD_652_^71^ and [PC] = (OD_615_ − 0.474 × OD_652_)/5.34^72^, respectively.

### Confocal imaging of lectin-labeled cells

Cells of the PAL control and the Paleoproterozoic model cultures were gently pelleted by centrifugation at 1000 × *g* and resuspended in (4-(2-hydroxyethyl)-1-piperazineethanesulfonic acid buffer (10 mM, pH 7.5) supplemented with fluorescein-labeled wheat germ agglutinin (WGA) and concanavalin A (ConA) lectins (Vector Laboratories, Burlingame, CA) to a final concentration of 5 μg ml^−1^. Following gentle rocking at room temperature in the dark for 60 min, the cells were carefully rinsed with a buffer to remove unbound lectins and imaged with a Zeiss 710 laser scanning confocal microscope, using 488 nm (Ar) and 594 nm (HeNe) laser lines. Unstained *S*. 8806 cells had no green autofluorescence when excited with 488 nm laser.

### Electron microscopy imaging

The SEM was performed on Millipore filters (Millipore, Billerica, MA) or silicon wafer (Agar Scientific Ltd, Stansted, UK) by secondary electrons using a Hitachi S5000 scanning electron microscope (Hitachi High Technologies America Inc, Pleasanton, CA). The cells were fixed with 2% glutaraldehyde, post-fixated with 1% OsO_4_, dehydrated with graded ethanol series, critical point dried using the Tousimis AutoSamdri 815 Critical Point Dryer (Tousimis, Rockville, MD), and sputter coated with gold-palladium using a Tousimis Sputter Coater. For transmission electron microscopy (TEM), samples were high-pressure frozen using a Leica EM PACT2 (Leica Microsystems Inc, Buffalo Grove, IL) and freeze-substituted using a Leica EM AFS2. Dehydration with acetone was followed by stepwise infiltration with epoxy resin and heat polymerization. Resin blocks were sectioned using a Leica EM UC6 microtome. Samples were stained with 2% uranyl acetate and lead citrate and imaged using an FEI Tecnai 12 transmission electron microscope (FEI, Hillsboro, OR).

### SEM-coupled EDS analysis

The cells were fixed with 2% glutaraldehyde, applied on poly-l-lysin covered silicon wafer, and let settle for 30 min. Seawater was then removed and the cells were briefly washed twice with double-distilled water (DDW), air-dried, and desiccated. The SEM-coupled EDS analyses were performed on an FESEM Gemini Ultra 55 electron microscope (Carl Zeiss Microscopy, Thornwood, NY) coupled with EDS system for elemental X-ray analysis. Map and line-scan spectra were collected at 10 keV with a dwell time of 10 s.

### ζ-potential assay

The ζ-potential was determined by measuring directional mobility of the cells in an electric field. Cells of the PAL control and the Paleoproterozoic model cultures were gently pelleted by centrifugation at 1000 × *g* and resuspended either in ice-cold DDW, or 10 mM of ice-cold NaCl, CaCl_2_, or MgCl_2_ solutions (pH 8.0 for all) to OD_750_ ∼0.1. Integrity of the cells was confirmed microscopically. Electrophoretic mobility of cells was measured at +4 °C using Zetasizer Nano ZS (Malvern Instruments Ltd, Malvern, UK), with ten measurements of ten reads each collected for each sample. The ζ-potential was automatically calculated from the experimental mobility data using the Smoluchowski’s equation.

### Ion adsorption experiments

Three batches of gently pelleted cells from the PAL control and the Proterozoic model cultures were suspended in 450 mM NaCl, 10 mM NaN_3_, and 10 mM EDTA to inactivate cells and chelate divalent cations. After 30 min incubation, the cells were washed twice with 450 mM NaCl, suspended in 450 mM NaCl solution containing 5 mM CaCl_2_ and 25 mM MgCl_2_, and incubated with gentle rocking at room temperature for 30 h. The cells were then removed using a pre-combusted Whatman GF/A glass microfiber filter and Ca and Mg concentrations in the filtrate were determined with Varian 720-ES ICP-OES (Varian Inc., Walnut Creek, CA). Ash-free dry weight was calculated by subtracting the weight of ash remaining after biomass combustion at 500 °C for 5 h from the dry weight of cells dehydrated at 60 °C overnight.

### Nucleic acids extraction

For genomic DNA (gDNA), 20 ml cells from the PAL control culture were collected by centrifugation (8000 × *g* for 8 min at +4 °C). For RNA, 50 ml cells were collected from mid-log phase cultures of the PAL control and the high Proterozoic model. The gDNA was extracted using Wizard^®^ Genomic DNA Purification kit (Promega Co, Madison, WI). The total RNA was extracted with Trizol reagent (Invitrogen Ltd, Carlsbad, CA), following the standard protocol. Any contaminating DNA was digested with DNase I (Ambion, Austin, TX), and ribosomal RNA was depleted using RiboMinus kit (Invitrogen). RNA quantity was determined with Qubit DNA quantification system and high sensitivity assay reagents (Invitrogen) and quality was determined using a Bioanalyzer 2100 (Agilent Technologies, Santa Clara, CA).

### Library preparation and sequencing

Illumina-compatible paired-end libraries for DNA and RNA were prepared using PrepX™ DNA and RNA library kits on fully automated Apollo 324™ System (IntengenX, Pleasanton, CA), quantified with qPCR using the Kapa Illumina Library Quant kit on a LightCycler 480II Thermal Cycler (Roche Applied Science, Indianapolis, IN) and pooled in an equimolar ratio. Two lanes of HiSeq2000 v3 chemistry were run with paired-end 100 cycle reads. Demultiplexing and fastQ files generation were performed using the Illumina CASAVA (v1.8) software suite and generated 7 × 10^7^ read pairs.

### Genomic and transcriptomic data analyses

Raw data cleaning, trimming, and pairing were performed with Trimmomatic 0.22^73^ and yielded 2.8 × 10^6^ paired reads. Fifty-six genomic contigs were assembled using Velvet assembler^74^ (*k* = 49) and Glimmer^75^-predicted open reading frames (ORFs) were aligned against Pfam^76^ version 23 using HMMER^77^ version 3.0 to identify protein motives. Protein functions were assigned to the ORFs using tblastn search (blast v.2.2.22) against the non-redundant GenBank database provided by the National Centre for Biotechnology Information (NCBI) database. The RNA reads (44 × 10^6^ for the PAL control and 56.5 × 10^6^ for the high Proterozoic model) were mapped against the genomic contigs using Bowtie2^78^ version 2.1.0 (89% and 94% alignment rate for the PAL control and the high Proterozoci model, respectively) and the number of reads mapped to each ORF was counted using the htseq-count tool in version 0.5.3 of the HTSeq Python package^79^. The expression level was normalized to the total number of reads^40^.

### Data availability

The data that support the findings of this study are available from Open Science Framework at https://osf.io/3cu58^80^.

## Electronic supplementary material

Supplementary Information
