# Supplementary material for: High pCO2-induced exopolysaccharide-rich ballasted aggregates of planktonic cyanobacteria could explain Paleoproterozoic carbon burial
Source: Nat Commun. 2018 May 29;9:2116. doi: 10.1038/s41467-018-04588-9 (PMC5974010; doi:10.1038/s41467-018-04588-9)
Supplement: Supplementary file 1 — Supplementary Information [file 41467_2018_4588_MOESM1_ESM.pdf]

Supporting Materials for

High pCO$_2$-induced exopolysaccharide-rich ballasted aggregates of planktonic cyanobacteria could explain Paleoproterozoic carbon burial

Kamennaya et al

This file contains:

Supplementary Figures 1 – 3

Supplementary Tables 1 – 3

Supplementary Figures:

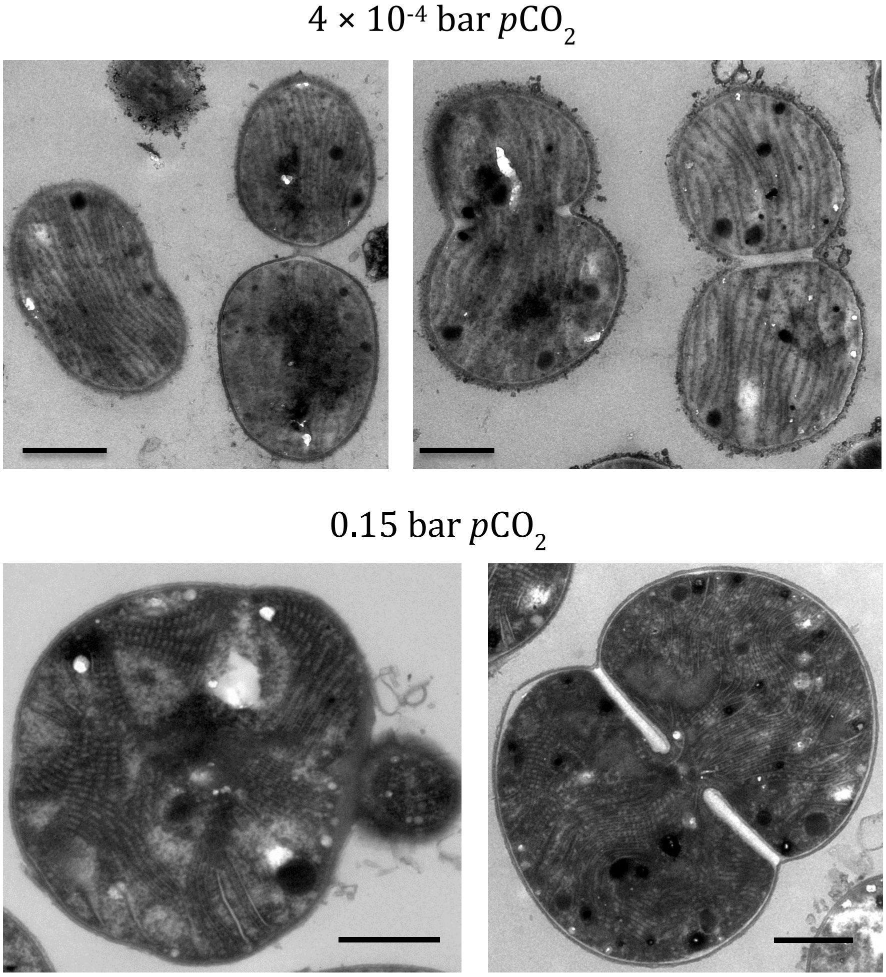

Supplementary Figure 1. Transmission electron microscopy (TEM) of cells from the PAL control and the high Paleoproterozoic model cultures. The TEM micrographs show tight arrangement of the thylakoid membranes bearing densely packed light-harvesting phycobilisome complexes in the Proterozoic model cells as compared to the looser thylakoid membranes arrangement in cells of the PAL control culture. Scale bar = 1 μm.

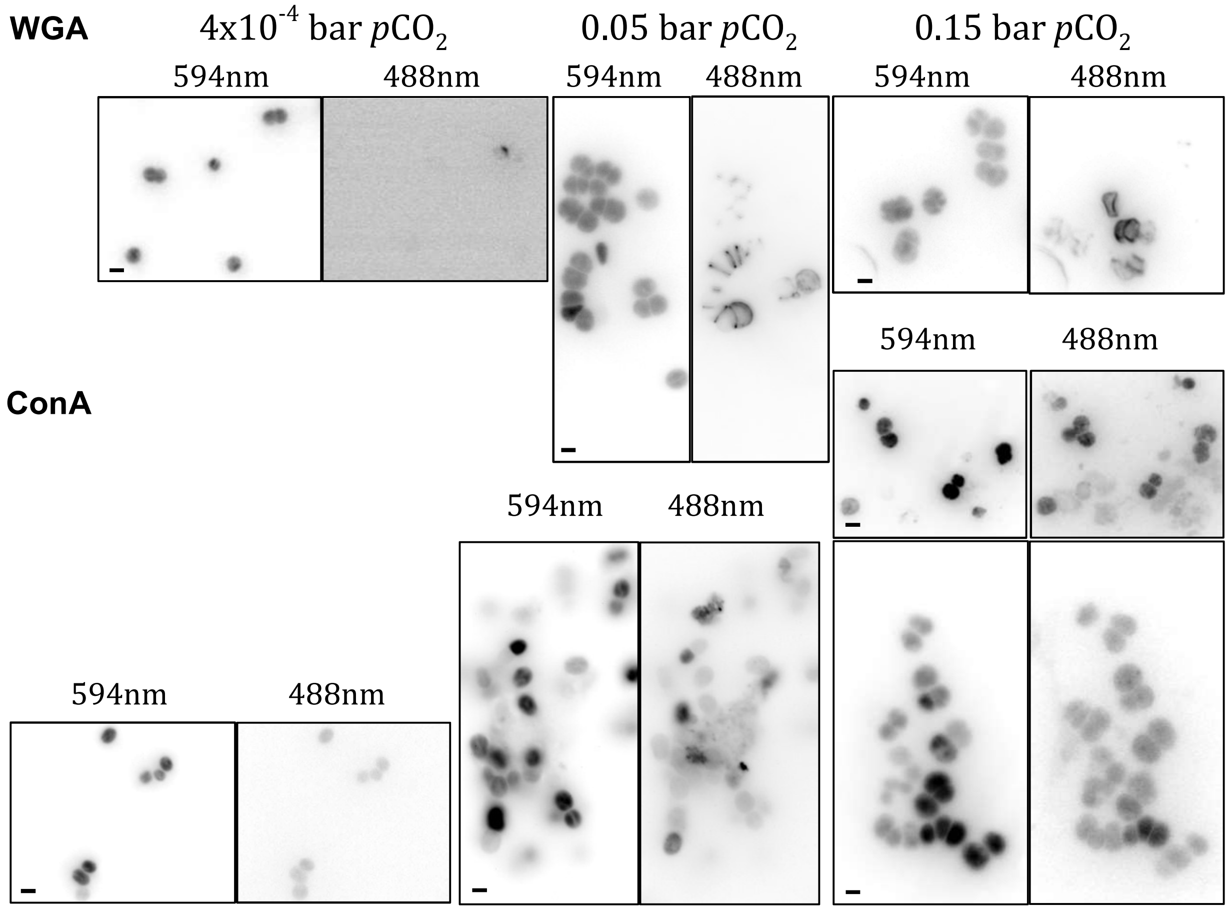

Supplementary Figure 2. Single line laser scanning confocal images of EPS-stained cells from the PAL control and the Proterozoic model cultures. The cyanobacterial cells on the left (chlorophyll autofluorescence; 594 nm laser line, Texas Red filter) and EPS stains on the right (WGA and ConA lectins; 488 nm laser line, FITC filter) shown as two separate images reveal very little EPS stain signal for the PAL control cells but dramatic accumulation of EPS in cell aggregates from the Proterozoic model cultures. Unstained *S.* 8806 cells had no green autofluorescence when excited with 488 nm laser. Bar scale = 1 μm.

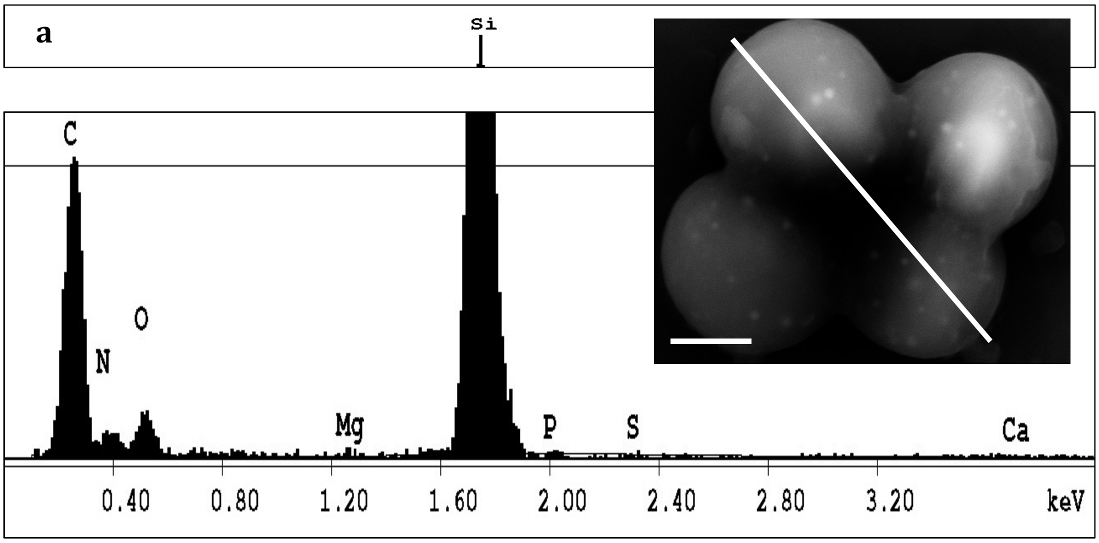
a
Si
C
N
O
Mg
P
S
Ca
0.40
0.80
1.20
1.60
2.00
2.40
2.80
3.20
keV

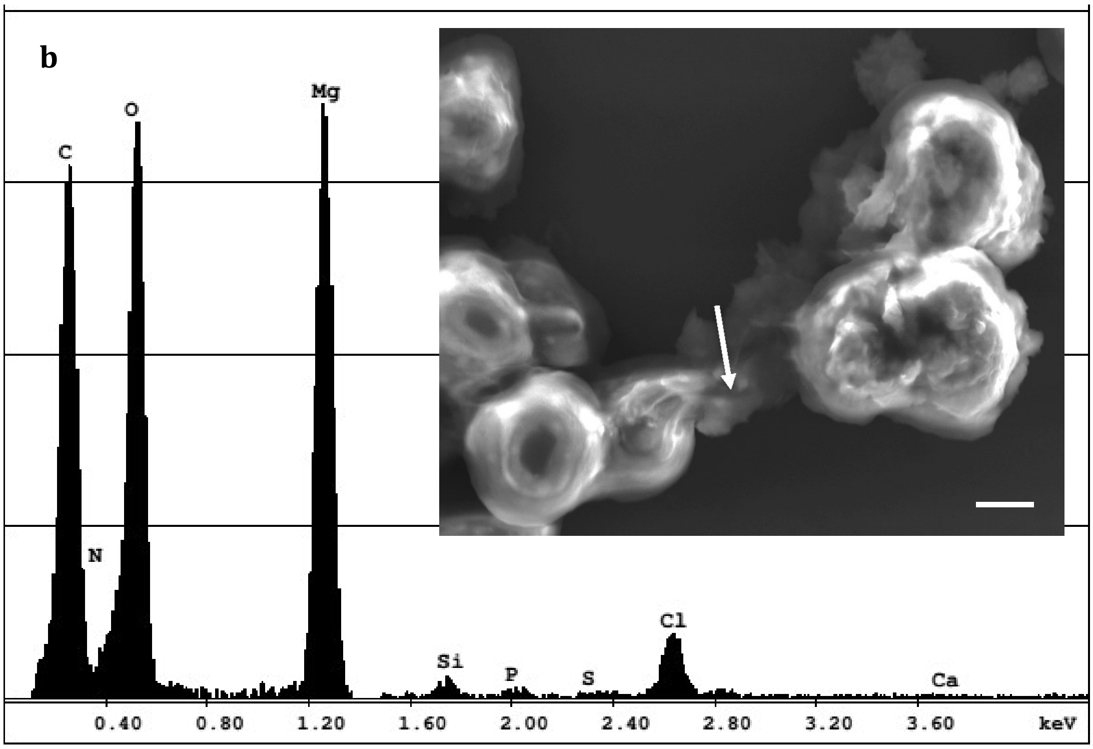
b
C
O
Mg
N
Si
P
S
Cl
Ca
0.40
0.80
1.20
1.60
2.00
2.40
2.80
3.20
3.60
keV

c

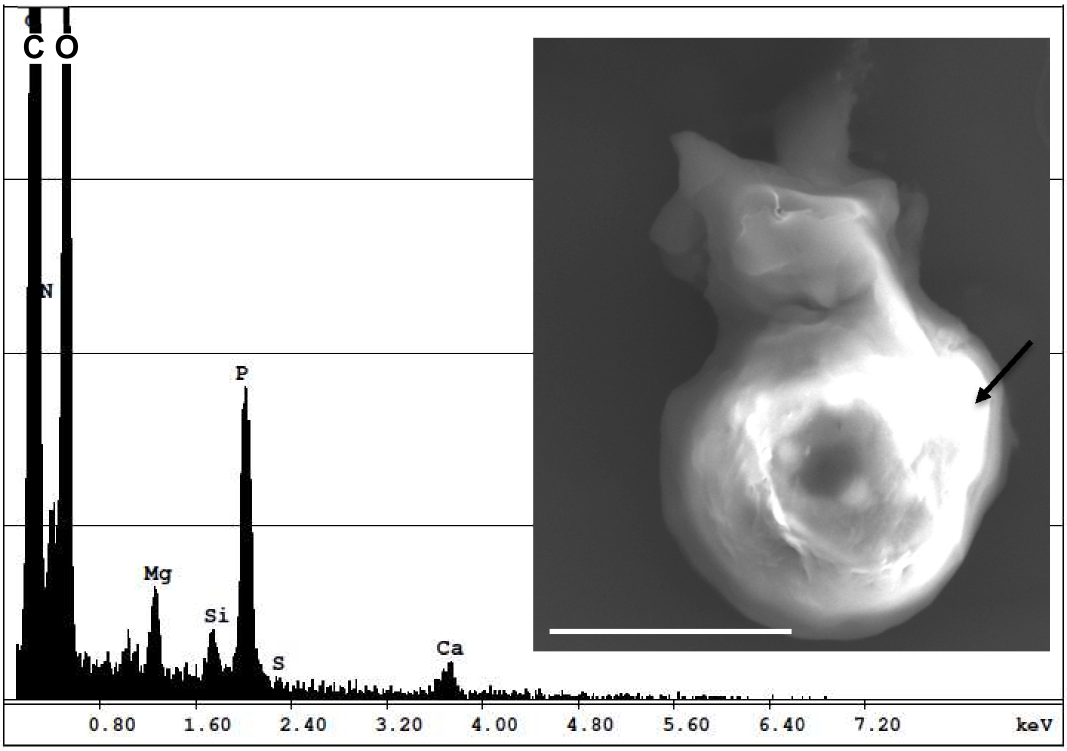

Supplementary Figure 3. Elemental spectra from SEM-coupled EDS analyses of materials from the PAL control and the Paleoproterozoic model cultures. Representative results from line-scan (a) and point-location (b, c) cumulative EDS elemental spectra of the PAL control cells (a) and the EPS material in aggregates of the high Proterozoic model cells (b, c) show accumulation of Ca and Mg cations in EPS of the Proterozoic cells as compared to cells of the PAL control. High Cl and P signals that coincide with Ca and Mg signals imply presence of Ca/Mg chlorides and phosphates in EPS of the Proterozoic cells. The Si signal originates from the Si wafer substrate. Scale bar = 1 μm.

Supplementary Tables

Supplementary Table 1

Probability values (*p*) and sample sizes (*n*) for unpaired two-sample *t*-test analyses applied to test similarity between the characteristics of *S.* 8806 cells cultivated under different *p*CO_2

	Cell diameter	SGR	Chl *a*	PC	Ca sorption	Mg sorption
p, PAL/LP	0.863	0.179	*0.027*	*0.017*	*0.021*	*0.007*
p, PAL/HP	0.694	0.773	*0.013*	*0.012*	*0.005*	*<0.001*
n	12	4	3	3	3	3

Cell diameter, specific growth rate (SGR), cellular content of chlorophyll *a* (Chl *a*) or phycocyanine (PC) and the amount of absorbed Ca or Mg cations were compared between cells from the PAL control (PAL) vs the Low Proterozoic (LP) or the High Proterozoic (HP) model cultures. The difference was considered significant when $p<0.05$ (in italic).

Supplementary Table 2

Probability values (*p*) for unpaired two-sample *t*-test analyses applied to test similarity between ζ-potential values measured for *S.* 8806 cells cultivated under different *p*CO_2

	DDW	NaCl	$CaCl_2$	$MgCl_2$
p, PAL/LP	*<0.001*	0.327	0.563	0.850
p, PAL/HP	*<0.001*	*0.004*	0.051	0.070

The ζ-potential values were compared between *S.* 8806 cells from the PAL control vs the Low Proterozoic (LP) or the High Proterozoic (HP) model cultures. The cells were

dispersed in DDW or in NaCl (10 mM), $CaCl_2$ (10 mM) or $MgCl_2$ (10 mM) solutions. The difference was considered significant when $p<0.05$ (in italic).

Supplementary Table 3

Amount of Ca and Mg cations adsorbed within 30 h by 1 mg of *S.* 8806 cells[1] from the PAL control or the Paleoproterozoic model cultures

Treatment (pCO_2 bar)	Ca^{2+} (μg)	Mg^{2+} (μg)
PAL control (4×10^{-4})	0.35 ±0.04	1.04 ±0.04
Low Proterozoic (0.05)	0.41 ±0.05	1.23 ±0.13
High Proterozoic (0.15)	0.47 ±0.02	1.66 ±0.11

[1]Ash-free dry weight

The range given is ± s.d., *n* = 3.
